# Cephalometric changes in pharyngeal airway dimensions after functional treatment with twin block versus myobrace appliances in developing skeletal class II patients: a randomized clinical trial

**DOI:** 10.1186/s12903-023-03701-9

**Published:** 2023-12-13

**Authors:** Ahmed M. Madian, Dina Elfouly

**Affiliations:** Department of Orthodontics, Faculty of Dentistry, Alexandria University, Champollion St., P.O. Box 21521, Azarita, Alexandria, Egypt

**Keywords:** Class II, Twin block, Myobrace, Myofunctional appliance, Sagittal pharyngeal Airway Dimension

## Abstract

**Background:**

Several appliances have been used for correction of developing skeletal Class II, including different myofunctional appliances as Twin block (TB)as well as the new pre-fabricated Myobrace (MB) appliance. However, the effects of these devices on the pharyngeal airways have not been compared in the literature. Thus, the aim of this study was to compare the effects of two Class II correction appliances; TB and MB on the sagittal pharyngeal airway dimension (SPAD), including the nasopharyngeal airway area (NPAA), the oropharyngeal airway area (OPAA), and the laryngopharyngeal airway area (LPAA).

**Methods:**

This is a two parallel arms randomized comparative clinical trial. Twenty-six children of 9–12 years with Skeletal Class II malocclusion due to mandibular deficiency and normal maxillary growth as confirmed by lateral cephalometric X-ray readings (ANB angle > 4° and SNB angle < 78) and Cervical vertebral maturational index (CVMI) 1 or 2 were randomly assigned into two equal groups. Group I: TB, Group II: MB (prefabricated functional appliance, Myofunctional Research Co., Australia). Lateral cephalograms were taken for all patients in both groups before treatment (T1) and after treatment (6 months later) (T2). The primary aim was to assess pre and post treatment changes in the SPAD in each group, and compare between the two study groups. The secondary aim was to evaluate the sagittal skeletal measurements such as the SNA, SNB, ANB, Wits appraisal, as well as vertical skeletal measurements represented by the Frankfurt-mandibular plane angle (FMA) measured pre- and post-treatment. The independent samples t-test was used to compare the two study groups, and the mean difference and 95% confidence intervals (CI) were computed. The paired samples t-test was used to compare various parameters between T1 and T2 within each group. The cutoff for significance was *p*-value < 0.05. Data were analyzed using IBM SPSS for Windows (Version 26.0).

**Results:**

By Comparing changes in airway measurements within each group, it was found that NPAA, OPAA, and LPAA increased significantly after treatment within each group of MB and TB. TB group showed significantly higher mean difference (T2-T1) in both NPAA and OPAA than MB group with 28.39 (± 56.75) and 40.46 (± 52.16) respectively. The increase in LPAA values was not statistically significant at (T2-T1) between both groups. Regarding skeletal changes, there was a significant increase in the SNB values between T1 and T2 within each group with 2.82 (± 3.32) for MB group and 3.79 (± 3.06) for TB group Moreover, there was a significant decrease in the ANB values between T1 and T2 within each group by 2.42 (± 2.70) for MB group and 3.06 (± 1.14) for TB group. Similarly, there was a significant decrease in the ANB values between T1 and T2 within each group by -2.13 (± 0.62) for MB group and − 2.46 (± 0.72) for TB group. No significant differences were found between both groups in SNA, SNB, ANB and Wits appraisal at *p* = 0.06, *p* = 0.45, *p* = 0.43 and *p* = 0.22 respectively. FMA did not show significant difference between T1 and T2 within each group, nor showed a significant mean difference between both groups at T2-T1.

**Conclusions:**

TB was more effective than MB in improving the upper (NPAA) and middle (OPAA) airways, while no difference was found regarding the lower airway (LPAA). Both TB and MB reduced the severity of developing skeletal class II due to mandibular retrognathism by forward posturing of the mandible. Thus, patients with airway problems would benefit more from TB than MB.

## Introduction

Skeletal Class II malocclusion is considered one of the most common dentofacial anomalies affecting almost one-third of the population [1]. It occurs either due to maxillary prognathism, mandibular retrognathism or a combination of both. Mandibular retrognathism has been reported to be the most common cause [2, 3].

As a result of mandibular retrognathism, the space between the cervical column and the mandibular corpus is diminished, the tongue and soft palate are posteriorly postured; consequently, narrowing the airway dimensions [4]. Thus, the pharyngeal airway dimensions were found to be decreased in Angle Class II division 1 patients [5, 6]. It was proven that early diagnosis of skeletal Class II malocclusion is best treated with the use of functional appliances. These appliances allow the forward growth of the mandible and prevents upper airway collapse during sleep [7–10].

The Twin Block (TB) is one of the preferred removable functional appliances used in correcting retrognathic mandible in developing Class II malocclusion patients [7]. This appliance increases pharyngeal airway dimensions through the forward movement of the mandible and hyoid bone [7, 11–16]. In 1980s, prefabricated functional appliances (PFAs) have been introduced with a number of various brands (such as Myobrace, and Occlus-o-Guide) providing them [17].

Soft, non-customized PFAs are often utilized in combination with myofunctional training, setting them apart from traditional functional appliances. In contrast to other kinds of full-time functional appliances (e.g., Twin Block, Herbst), they are only worn part-time (like the conventional ‘activator’). Clinical research comparing the effects of PFAs to conventional functional appliances has just been published recently, despite the fact that PFAs have been used for decades [18–20]. Prefabricated myofunctional devices aim at correcting the etiological factors of malocclusion through the elimination of dysfunction in the orofacial muscle activity, tongue posture, and improving the airway volume, enhancing the occlusion [21, 22] in developing Class II patients.

The Myobrace (MB) is a ready-made orthodontic appliance employed for correcting malocclusions in children and teenagers with late mixed dentition [23]. This appliance retains the tongue’s position and rebalances the face and masticatory muscles [24]. The myofunctional impact, dental alignment, and mandibular growth are the three goals of this appliance. It’s constructed on an edge-to-edge incisal relationship between the two arches and comprises of a single block. The only structural difference, compared to the other “Trainer System” appliances, is the internal additional hard nylon element, called “Inner-Core”, or “Dynamicore” [25]. Myobrace’s two-material technology increases patient compliance, and the device’s myofunctional re-education capabilities allow the tongue and lips to continue aligning the teeth even after treatment has ended [26].

Lateral cephalometry, with its routine use, cheap cost, minimum exposure to radiation, and enough information provided regarding the pharyngeal airway compared to those obtained from three-dimensional cone-beam computed tomography (CBCT), is the recommended approach in this study [27, 28].

Although the efficiency of the functional appliances to increase the airway dimensions has been widely investigated in the literature, none of the previous studies have compared the effect of TBA and Trainer for kids (MB) on the pharyngeal airway during the pubertal growth period, throughout a prospective clinical trial. Thus, our goal with this research was to evaluate the relative effectiveness of the Twin-block versus MB appliances in improving the sagittal pharyngeal airway dimension (SPAD) in adolescents having skeletal Class II malocclusion with retrognathic mandible, through a randomized clinical trial. Moreover, sagittal and vertical skeletal changes accompanying each of the tested appliances were assessed. The null hypothesis of this study there is no difference between the studied appliances regarding their effect on the SPAD.

## Materials and methods

### Study Design

This is a two parallel arms randomized comparative clinical trial, involving two groups, each evaluating one of the tested Myofunctional appliances. This study followed the CONSORT guidelines of reporting of randomized controlled trials [29].

**PICO**

**Table:** 

**Patient**	Growing skeletal Class II patients with mandibular deficiency
**Intervention**	Myobrace functional appliances.
**Control**	Using Twin block functional appliances.
**Outcomes**	Evaluate the pharyngeal airway changes in both interventions.

### Participants

Children were selected from the outpatient clinic of the Orthodontic Department, Faculty of Dentistry, Alexandria University.

#### Inclusion criteria

1. Healthy children age ranged from 9 to 12 years.
2. Skeletal Class II malocclusion with mandibular deficiency and normal maxillary growth depending on clinical diagnosis and confirmed with the lateral cephalometric X-ray readings (ANB angle > 4° and SNB angle < 78).
3. Cervical vertebral maturational index (CVMI) 3 assessed by lateral cephalograms [30].

#### Exclusion criteria

1. Previous orthodontic/orthopedic treatment.
2. Previous extractions.
3. Mandibular shifts.
4. Severe crowding.
5. Anterior open bite.
6. Any peri-oral habits.

### Sample size calculation

The sample size was planned based on 95% confidence level to detect differences in SNB angle between Twin block and Myobrace appliances in class II malocclusion patients. Johnson et al. [31] reported mean ± SD difference in ANB angle after using Twin block appliance and after Myobrace appliance = 2.20 ± 1.22 and 1.14 ± 1.33 respectively. The calculated mean ± SD difference between both groups = 1.06 ± 1.28 and 95% confidence interval= -0.14, 2.26. Forward growth of the mandible using functional appliances is assumed to improve the pharyngeal airway dimensions [12]. The minimum sample size was calculated to be 12 per group, increased to 13 to make up for cases lost to follow up. The total required sample size = number of groups × number per grou*p* = 2 × 13 = 26 [32]. This was calculated using MedCalc Statistical Software version 19.0.5 [33].

### Ethical approval

This study was approved by the Institutional Review Board at the Faculty of Dentistry, Alexandria University, Alexandria, Egypt (IRB:00010556–IORG:0008839) Manuscript Ethics Committee number (0418-03/2022). The trial was registered on ClinicalTrials.gov, with the name of the registry being “CEPHALOMETRIC CHANGES IN PHARYNGEAL AIRWAY DIMENSIONS AFTER FUNCTIONAL TREATMENT WITH TWIN BLOCK VERSUS MYOBRACE APPLIANCES IN DEVELOPING SKELETAL CLASS II PATIENTS: A RANDOMIZED CLINICAL TRIAL.” The trial registration number is NCT05610150 on 09/11/2022, and the URL is https://clinicaltrials.gov/ct2/show/NCT04926389. All patients were informed of the procedure and signed informed consents accordingly. All research procedures were performed in accordance with the relevant guidelines and regulations, as stated in the Declaration of Helsinki. Informed consent was obtained from all subjects/or their legal guardian(s) for the use of their records.

### Randomization and allocation concealment

**Fig. 1:**
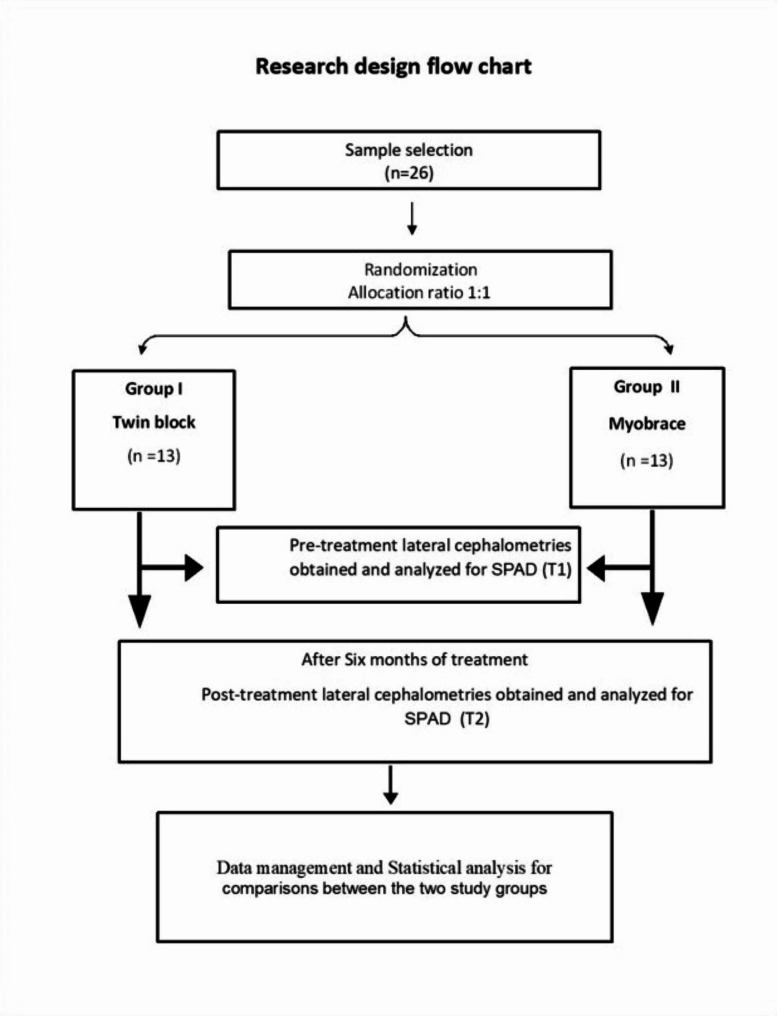
Research design flow chart

Twenty-six children were randomly assigned in a 1:1 ratio using a computer-generated list of random numbers [34] to one of the two groups; Group I: TB group, Group II: MB group (prefabricated functional appliance, Myofunctional Research Co., Australia). The allocation sequence was concealed from the researcher and the patients. When a patient was deemed as eligible for enrollment, the patient was assigned to a treatment group using opaque and sealed envelopes containing the allocation number [35]. A research design flowchart is represented in (Fig. 1), summarizing the study procedures.

### Blinding

Due to the nature of the intervention, it was not possible to blind the patients or the orthodontist. The researcher and the statistician who evaluated the data were blinded.

## Methods

### Appliance fabrication

#### Group I: Twin block group

Wax bite registration included advancing the mandible of each patient until the upper and lower central incisors were in an edge-to-edge relation and a gap of 2–3 mm beyond the freeway space. Depending on the degree of the malocclusion, the procedure included one or two stages of advancement. The appliance should be worn at all times except for eating, as per the instructions [36]. Six months of follow-up were conducted at 4-weeks intervals.

#### Group II: Myobrace group

The appropriate size of Myobrace is chosen by using a special ruler to measure the distance between the distal portion of the lateral upper right incisor and the left, regardless of any crowding or diastema. The measure is based on the mesial-distal dimensions of the upper incisors, and not on their position. In cases where there is a severe crowding or wide spaces, and it is difficult to make measurements with a ruler, they can be measured individually and them added together, to get the total size of the four upper incisors. This distance is them confronted with a special table to choose the correct size of MB [37]. If the choice falls between two sizes, it is preferred to choose the larger one. Once chosen and inserted in patient’s mouth, upper canines, even if not yet erupted, must be in their slots, so that the dental mildines coincided with the appliance’s midline [38]. Patients were instructed to use the device throughout the day for an increasing amount of time each day during the first week. Patients were told to use the appliance overnight for at least 8 h beginning by the second week. Following the manufacturer’s instructions, patients wore their appliances for a minimum of 1–2 h per day and overnight beginning at the end of the first 4 weeks of therapy and continuing throughout the treatment period [39].

Patients of both groups were followed up for six months period for retention.

### Lateral cephalograms analysis

Lateral cephalograms were taken for all patients in both groups before treatment (T1) and after treatment (6 months later) (T2). Lateral cephalograms were taken using a standardized technique with the same machine; patients stood in the natural head position (NHP) [40, 41] and natural tongue posture with the teeth in centric occlusion. Patients were instructed to stand still and not to move their heads nor swallow during exposure. Lateral Cephalograms were compared between (T1) and (T2) for analysis of sagittal pharyngeal airway changes as well as skeletal measurements alterations in both intervention groups.

**Fig. 2:**
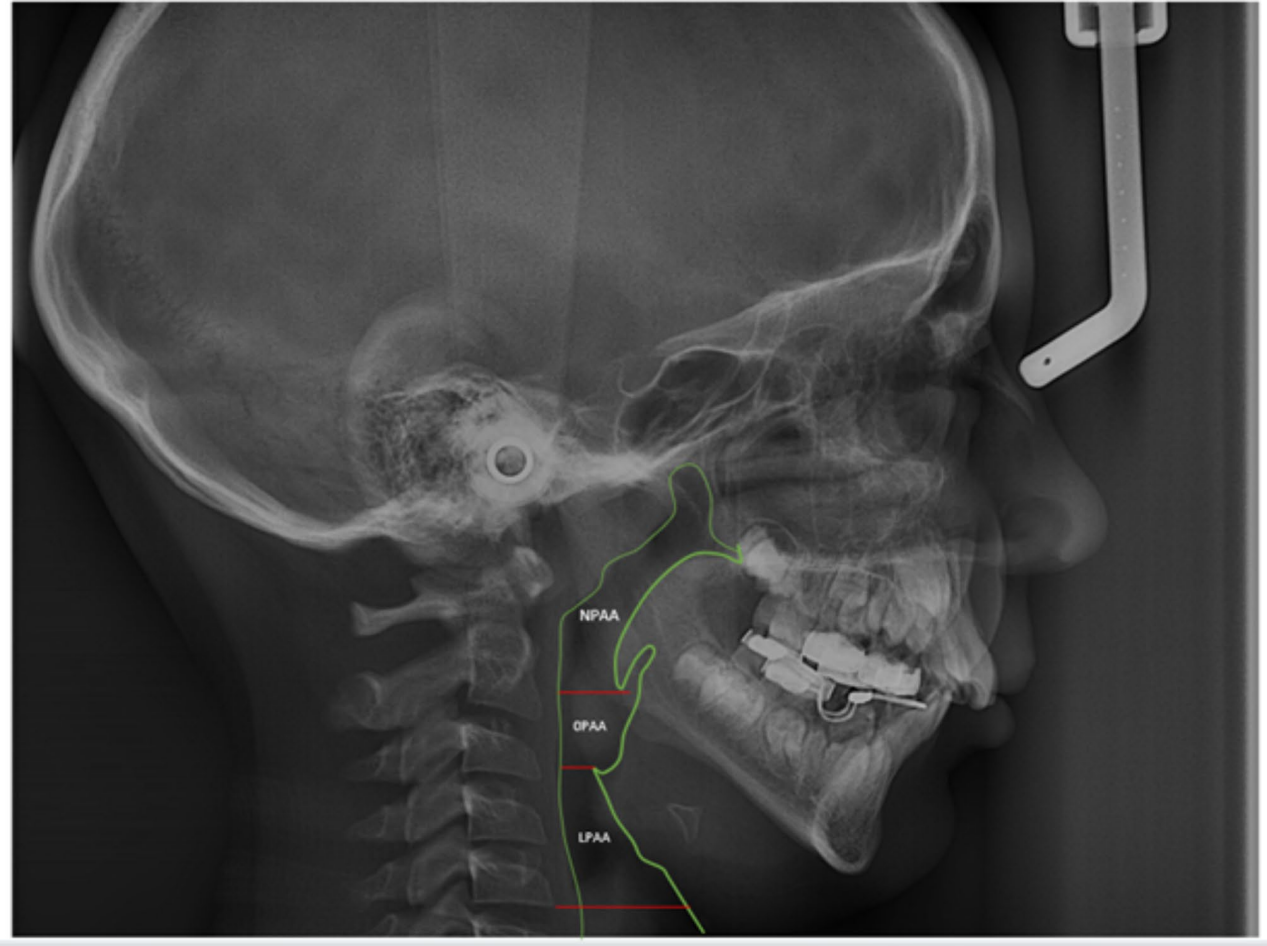
Cephalometric sagittal pharyngeal airway area measurements used in the study. NPAA: Nasopharyngeal airway area, OPAA: Oropharyngeal airway area, LPAA: Laryngopharyngeal airway area

Digital tracing of the Lateral cephalograms was done using Osirix open-source software [38] and the following points/ planes and landmarks were identified:

A. Skeletal measurements: SNA, SNB, ANB, FMA. (Table 1)
B. Pharyngeal airway area measurement:
  The pharyngeal airway was divided into three distinct regions using several anatomical markers: the nasopharyngeal airway area (NPAA), the oropharyngeal airway area (OPAA), and the laryngopharyngeal airway area (LPAA) [42]. A line drawn from the harmonium (H) to the posterior nasal spine (PNS) marked the highest limit of NPAA. A line drawn from the tip of the soft palate parallel to the Frankfort horizontal (FH) plane to the posterior wall of the pharynx delineated the NPAA’s lower limit. Differentiating between the OPAA and LPAA required drawing a line from the epiglottic tip at the level of the FH plane to the posterior wall of the pharynx. A line drawn parallel to the FH plane and going through the anteroinferior most point (C5AI) of the fifth cervical vertebra was used to establish the LPAA’s lower boundary. The same software was used to calculate the area. (Fig. 2)

**Table 1:** Skeletal measurements: SNA, SNB, ANB, FMA.

SNA	Angle between points Sella (S), Nasion (N) and A point and shows anteroposterior position of the maxilla relative to the anterior cranial base.
SNB	The angle between points Sella (S), Nasion (N), and B point and describes anteroposterior position of the mandible relative to the anterior cranial base.
ANB	Angle between points A, Nasion (N) and B point indicating the skeletal relationship between the maxilla and the mandible.
Wits appraisal	The linear distance between the perpendicular projections of points A and B over the functional occlusal plane.
FMA	Angle between mandibular plane and Frankfort horizontal plane.

### Intra-examiner and inter-examiner reliability

After a wash-out period of 2 weeks, the same and another calibrated independent investigator remeasured the whole parameters of 14 randomly selected x-rays to test intra and inter-examiner reliability using Intraclass Correlation Coefficient (ICC) [43] and Dahlberg error [44] for each airway measurement showing excellent agreement (Table 2).

**Table 2:** Intra- examiner and inter-examiner reliability

	Intra-examiner (examiner 1 – examiner 2)		Inter-examiner	
	ICC	Dahlberg error	ICC	Dahlberg error
**NPAA**	0.8987–0.9021	0.12	0.8874	0.21
**OPAA**	0.8765–0.8943	0.22	0.8662	0.33
**LPAA**	0.8689–0.9100	0.15	0.8878	0.16

ICC: Intraclass Correlation Coefficient

### Statistical analysis

Normality was checked for all variables using descriptive statistics, plots (Q-Q plots and histogram), and normality tests. All variables showed normal distribution, so means and standard deviation (SD) were calculated, and parametric tests were used. Comparisons between the two study groups were done using independent samples t-test with calculation of mean difference and 95% confidence intervals (CI). Comparisons of different parameters between T1 and T2 within each group were done using paired samples t-test. Significance was set at *p*-value < 0.05. Data were analyzed using IBM SPSS for Windows (Version 26.0).

## Results

**Fig. 3:**
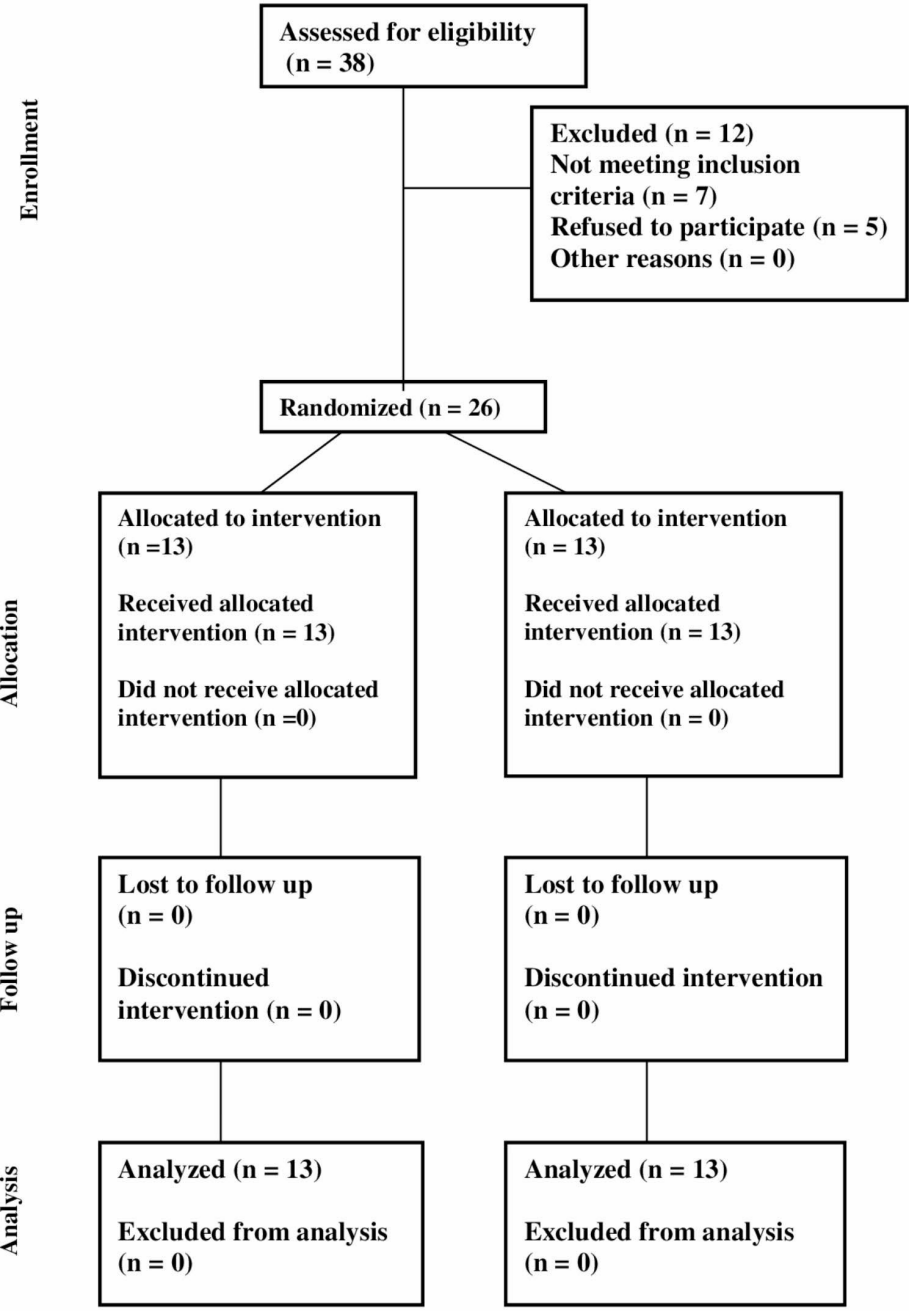
CONSORT Flow Diagram showing the patients’ flow throughout the clinical trial

Over the course of the study, there were no subject dropouts in the pre-intervention period, nor throughout the rest of the study. All the twenty-six initially recruited subjects completed the entire study period (13 subjects per group). The patient flow throughout the trial is presented through a CONSORT Flow Diagram (Fig. 3).

**Fig. 4:**
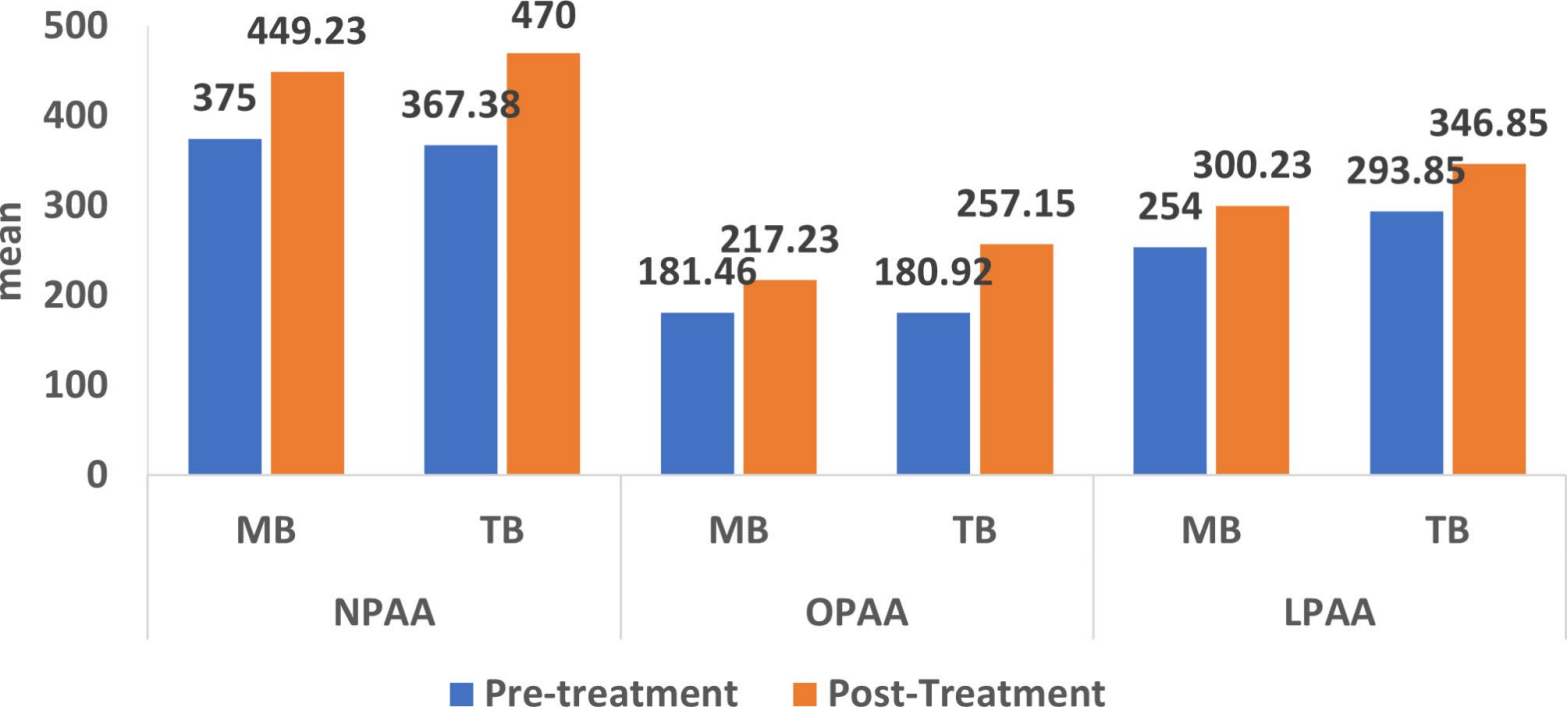
Intergroup comparison of the change in the skeletal cephalometric measurements

**Fig. 5:**
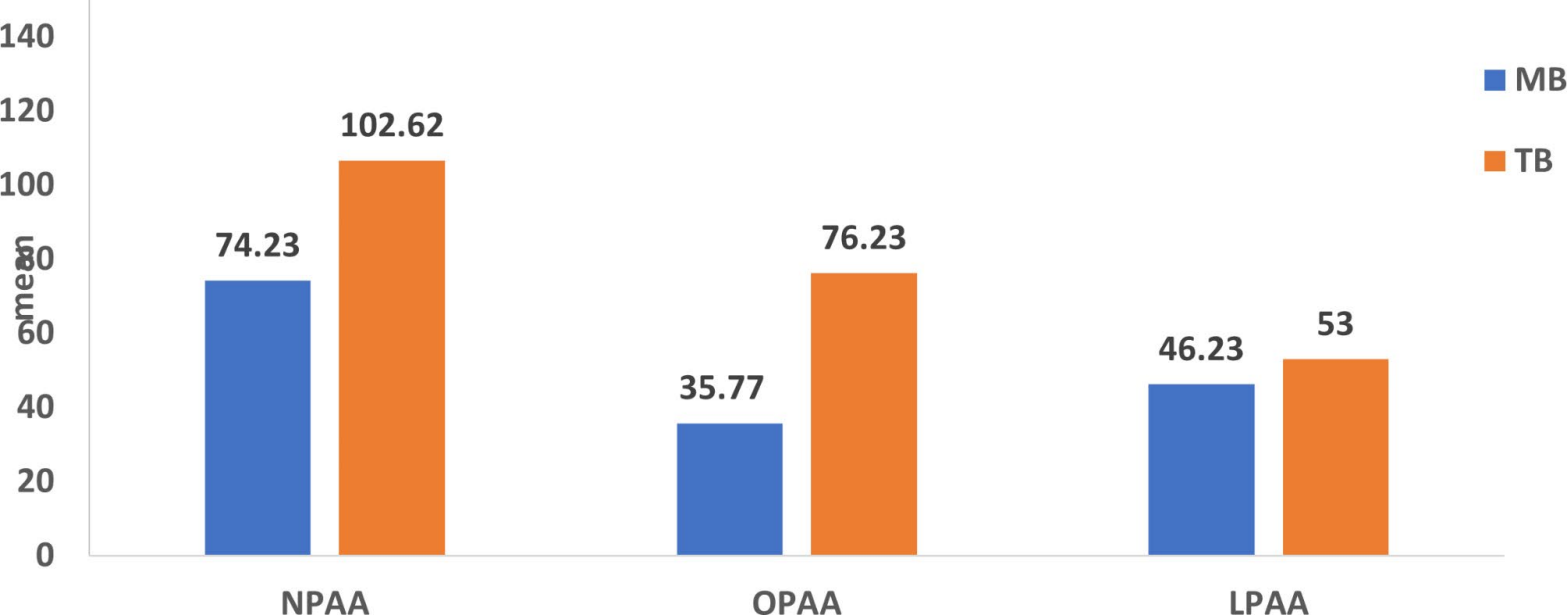
Intragroup comparison of the pretreatment and post-treatment pharyngeal airway area measurements

By analyzing the sagittal pharyngeal airway area measurements, it was found that NPAA, OPAA, and LPAA increased significantly after treatment within each group of MB and TB (*p* < 0.001) (Table 3; Fig. 4). The difference between the changes in the pharyngeal airway between both groups of MB and TB was analyzed. TB group showed significantly higher mean difference (T2-T1) in both NPAA and OPAA than MB group with 28.39 (± 56.75) at *p* = 0.02 and 40.46 (± 52.16) at *p* = 0.001 respectively (Table 3; Fig. 5). The increase in LPAA values was not significantly different at (T2-T1) between both groups with *p* = 0.33 (Table 3; Fig. 5). To ensure adequate study power, post-hoc power calculation was performed for all airway measurements (Table 3).

**Table 3:** Intergroup and Intragroup comparison of pharyngeal airway area measurements

		MB (n = 13)	TB (n = 13)	Difference	95% CI	*P* value	Post-hoc Power
		Mean (SD)					
**NPAA**	**T1**	375.00 (5.58)	367.38 (14.20)	7.62 (21.57)	-1.38, 16.61	0.09	40.87%
	**T2**	449.23 (37.67)	470.00 (22.09)	-20.77 (61.75)	-46.09, 4.55	0.10	37.71%
	**Difference**	74.23 (36.92)	102.62 (15.73)	-28.39 (56.75)	-51.95, -4.82	0.02*	68.9%
	***P value 2***	< 0.001*	< 0.001*				
**OPAA**	**T1**	181.46 (57.89)	180.92 (33.50)	0.54 (94.57)	-37.74, 38.82	0.98	5.10%
	**T2**	217.23 (60.68)	257.15 (44.99)	-39.92 (106.82)	-83.16, 3.32	0.07	44.80%
	**Difference**	35.77 (10.58)	76.23 (35.33)	-40.46 (52.16)	-62.38, -18.54	0.001*	**96.67%**
	***P value 2***	< 0.001*	< 0.001*				
**LPAA**	**T1**	261.68 (41.69)	293.85 (51.89)	-32.15 (94.13)	-70.25, 5.95	0.10	38.72%
	**T2**	300.23 (36.25)	346.85 (60.98)	-46.62 (100.35)	-87.72, -5.51	0.03*	**62.32%**
	**Difference**	38.54 (30.93)	53.00 (22.46)	-14.46 (54.04)	-36.35, 7.42	0.19	58.29%
	***P value 2***	< 0.001*	< 0.001*				

SD: Standard Deviation, CI: Confidence Interval, *P* value 1: Independent samples t-test, *p* value 2: Paired samples t-test. *: statistically significant at *p* value < 0.05

**Fig. 6:**
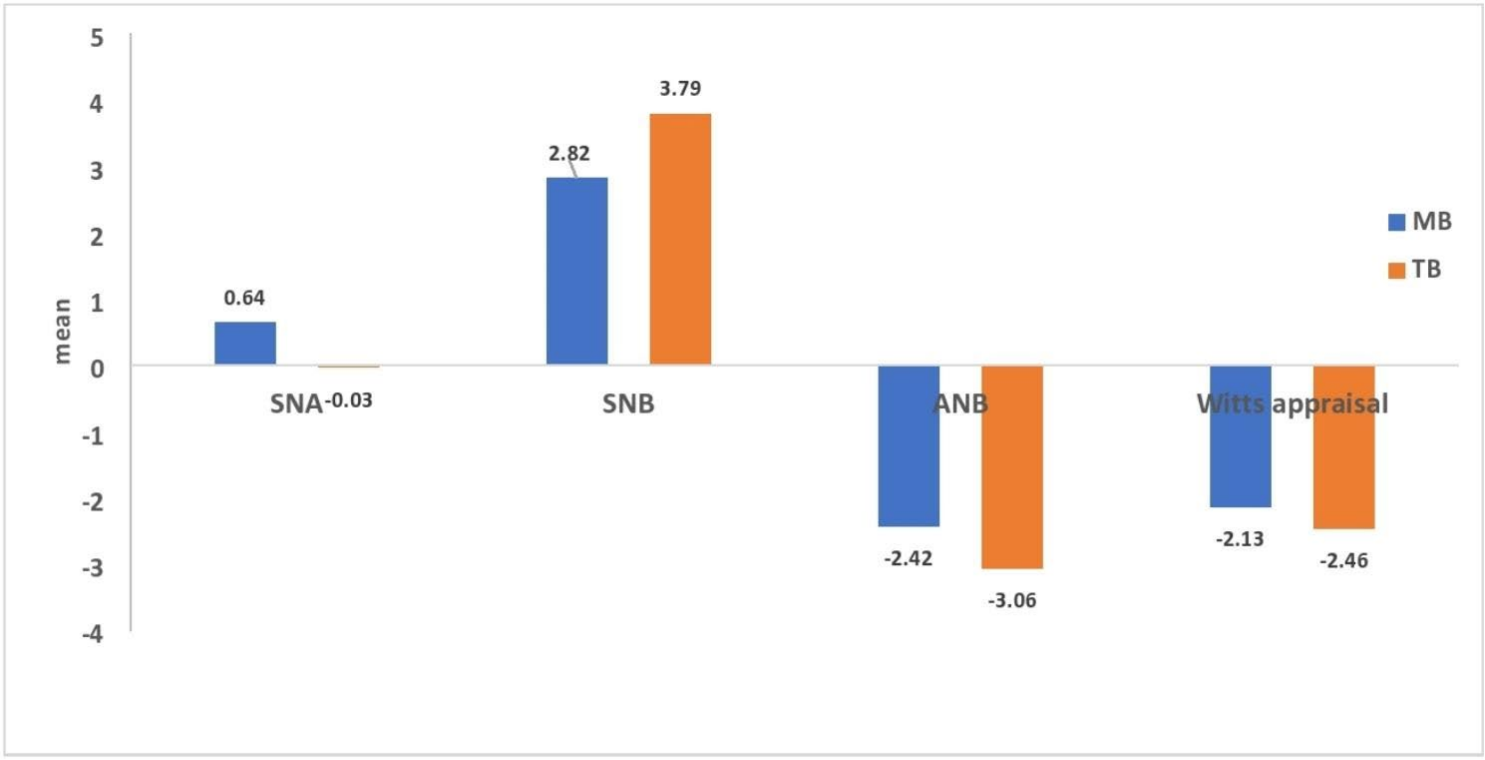
Intergroup comparison of the change in the pharyngeal airway area measurements

Regarding the skeletal angular readings within each group of MB and TB, the following results were found; there was no significant difference between SNA values at T1 and T2 within each group with *p* > 0.05 (Table 4). However, there was a significant increase in the SNB values between T1 and T2 within each group with 2.82 (± 3.32) at *p* = 0.01 for MB group and 3.79 (± 3.06) at *p* = 0.001 for TB group (Table 4). Moreover, there was a significant decrease in the ANB values between T1 and T2 within each group by 2.42 (± 2.70) at *p* = 0.007 for MB group and 3.06 (± 1.14) at *p* < 0.001 for TB group (Table 4). Similarly, there was a significant decrease in the ANB values between T1 and T2 within each group by -2.13 (± 0.62) for MB group and − 2.46 (± 0.72) for TB group (Table 4). No significant differences were found between both groups in SNA, SNB, ANB and Wits appraisal at *p* = 0.06, *p* = 0.45, *p* = 0.43 and *p* = 0.22 respectively (Table 4; Fig. 6). FMA did not show significant difference between T1 and T2 within each group, nor showed a significant mean difference between both groups at T2-T1 with *p* = 0.09 (Table 4).

**Table 4:** Intergroup and Intragroup comparison of cephalometric skeletal sagittal and vertical measurements

		MB (n = 13)	TB (n = 13)	Difference	95% CI	*P value 1*
		Mean (SD)				
**SNA**	**T1**	80.80 (3.04)	81.42 (1.38)	-0.62 (4.47)	-2.58, 1.33	0.51
	**T2**	81.44 (2.34)	81.39 (1.70)	0.05 (4.08)	-1.61, 1.70	0.96
	**Difference**	0.64 (1.08)	-0.03 (0.47)	0.67 (1.68)	-0.02, 1.36	0.06
	***P value 2***	0.06	0.82			
**SNB**	**T1**	72.82 (3.55)	71.69 (3.78)	1.13 (7.34)	-1.84, 4.10	0.44
	**T2**	75.64 (3.56)	75.48 (2.48)	0.16 (6.12)	-2.32, 2.65	0.89
	**Difference**	2.82 (3.32)	3.79 (3.06)	-0.97 (6.37)	-0.02, 1.36	0.45
	***P value 2***	0.01*	0.001*			
**ANB**	**T1**	8.00 (2.27)	8.97 (2.63)	-0.97 (4.90)	-2.95, 1.10	0.32
	**T2**	5.59 (2.47)	5.91 (1.97)	-0.32 (4.49)	-2.13, 1.48	0.72
	**Difference**	-2.42 (2.70)	-3.06 (1.14)	0.65 (4.13)	-1.03, 2.32	0.43
	***P value 2***	0.007*	< 0.001*			
**FMA**	**T1**	34.02 (3.93)	33.86 (6.66)	0.15 (10.93)	-4.27, 4.58	0.94
	**T2**	34.82 (3.96)	31.17 (3.01)	3.65 (7.04)	0.80, 6.50	0.01*
	**Difference**	0.80 (4.00)	-2.69 (5.96)	3.49 (10.15)	-062, 7.60	0.09
	***P value 2***	0.49	0.13			
**Witts appraisal**	**T1**	6.83 (1.83)	8.03 (2.05)	-1.20 (3.89)	-2.76, 0.38	0.13
	**T2**	4.70 (1.99)	5.57 (1.89)	-0.87 (3.88)	-2.44, 0.70	0.26
	**Difference**	-2.13 (0.62)	-2.46 (0.72)	0.33 (1.35)	-0.21, 0.88	0.22
	***P value 2***	< 0.001*	< 0.001*			

SD: Standard Deviation, CI: Confidence Interval, *P* value 1: Independent samples t-test, *p* value 2: Paired samples t-test. *statistically significant at *p* value < 0.05

## Discussion

According to literature, there is a reduction in pharyngeal airway dimensions in patients with skeletal Class II malocclusion and retrognathic mandible [5, 6, 45]. The retruded mandible is believed to lead to a more posterior tongue position, resulting in a decrease in pharyngeal airway capacity [45]. Despite the fact that many researches have analyzed the nature of skeletal class II correction by different functional appliances in developing children, few studies discussed the effect of these appliances on the sagittal pharyngeal airway dimensions (SPAD) [12, 45–49].

Thus, in the current study Skeletal class II patients with ANB angle greater than 4° and SNB angle less than 78° were given functional appliances in an attempt to improve their pharyngeal airway dimensions. Children chosen for this study were between the ages of 9 and 12 years, since previous researches have shown that the myofunctional appliances are most effective during the prepubertal stage of rapid growth [50]. This coordinates with Baccetti and McNamara study, where authors concluded that CVMI stages 3 and 4 represent the optimal treatment timing in dentofacial orthopedics [51, 52].

Although lateral cephalograms are not ideal for airway analysis, it was employed in this work due to its lower radiation dosage, availability and cost effectiveness [40]. In addition, the pharyngeal airway dimensions measured on lateral cephalograms were highly correlated with volumetric measurements obtained using 3-dimensional CT images [53]. Reproducibility of airway dimensions on lateral cephalograms was also proved to be of high accuracy [41].

When assessing pharyngeal airway parameters, most researches have exclusively used linear metrics [54]. However, sagittal airway area measurements correlate more strongly with 3D volumetric changes than linear measurements, as indicated by Aboudara et al. [55]. Thus, the present study utilized sagittal pharyngeal airway area measurements to compare the efficacy of the Twin-block versus MB appliances in the improvement of pharyngeal airway dimensions in adolescents with skeletal Class II malocclusion.

### Twin block group

The present study showed a statistically significant increase in the three airways (NPAA, OPAA, LPAA) between T1 and T2 after the use of TB appliance. This was in agreement with the results of previous researches [7, 9, 14, 42, 50, 56–60] which reported similar mean changes. On the contrary, there were different outcomes in a number of studies which showed that there were no differences in the pharyngeal airways after the application of TB appliance [7, 55, 60–63]. This variation between their results and present results could be due to the different airway analysis used.

From the present results, it was evident that the sagittal jaw relationship was significantly improved following TB treatment by increasing SNB and decreasing ANB and Wits appraisal. Similar results were obtained in earlier studies [7, 14, 31, 56, 61–64]. On the other hand, O’Brien et al. [65]. stated that the most important changes resulting from treatment in their study were dentoalveolar and that the statistically significant change in the skeletal relationship might not be considered clinically significant. These results might have occurred because measurements were taken at the start of the treatment and 15 months later, through which relapse might have taken place and caused diminished skeletal readings.

Regarding the vertical dimension assessed by FMA angle in this study, it showed no difference before and after treatment either within each group or between both groups. These results were similar to those obtained by previous studies [31, 61]; however, opposite results were obtained in other studies [7, 56, 62, 63]. This difference might be due to different patient age range or variable methods of measurements and different landmarks.

### Myobrace group

Unfortunately, there is a lack of evidence regarding the effect of MB on the airway dimensions. Therefore, this study was conducted regarding the use of MB for Class II patients to widen their airway passages. The current results agreed with AHN et al. [66] who confirmed the increase of oropharyngeal airway dimension after using MB appliance for the children with Obstructive Sleep Apnea Syndrome symptoms. Different results were obtained by Çoban et al. [64] who found statistically insignificant differences in pharyngeal and soft palate measurements after the use of MB appliance. These results could be justified as they measured upper airway only which is not believed to be affected by the retruded mandibular position [12]. In addition, linear measurements were taken in their study, which were shown to be inaccurate to represent the volumetric nature of airways when compared with the sagittal airway area measurements employed in the current study [50].

In this work, MB appliance postured the mandibular position forward by increasing SNB and consequently decreasing ANB and Wits appraisal. This was an agreement with earlier studies [31, 60, 63]. Oppositely, Çoban et al. [64] found that there was no significant change in the ANB angle following application of MB appliance although SNB angle was significantly increased in their study. Similar to the effect of TB on the vertical dimension of the face, MB caused insignificant change in the FMA angle. This was similar to the results of Çoban el al. [64] while different from another studies [31, 60].

### Twin block versus myobrace

There were no statistically significant differences between both groups regarding the sagittal skeletal relations with similar results obtained previously [31, 64]. Regarding the airway changes, TB was better than MB in expanding the NPAA and OPAA significantly. However, LPAA showed insignificant difference between both groups. On the contrary, both TB and MB appliances were previously reported to be ineffective in increasing pharyngeal airways [64]. These results could be explained by the fact that the design of the study was different, in addition they measured airways by linear parameters which have been shown to be inaccurate in expressing the 3D volumetric nature of airways [50].

Twin block was superior to MB because of the increased reciprocal force pushing backwards on the maxilla upon the practically full-time wear in combination with the exceptional retention of the TB. In addition, the TB was preferred by the patients because it did not impede their speech or daily activities [21, 22, 67, 68]. MB appliance looseness, particularly during sleep, and leaving a space between the teeth and the appliance resulting in poor compliance. Patients’ resistance to the MB appliance’s instructions resulted in extended delays and diminished effectiveness [18].

### Limitations of the study

Due to ethical concerns, this research did not include a control group that was not given any therapy in order to determine the skeletal treatment effects and airway modifications of both appliances compared to natural development. In addition, two-dimensional measurements on the lateral cephalometric radiographs cannot reveal the changes in the third dimension (transverse dimension of the airway). Finally, it is important to highlight that the short follow-up period of six months may be insufficient for evaluation, which also might have impacted the MB results, since it took most of the children two months to adapt to the appliance, achieving the required wear-time per day. To overcome this, Patients were observed for an additional six months after the trial period ended to assure the retentive period and prevent any relapses that could have occurred during the study period. At that stage, patients who needed further treatment were referred to the Orthodontic Department for fixed appliance therapy.

## Conclusion

TB was more effective than MB in improving the upper (NPAA) and middle (OPAA) airways, while no difference was found regarding the lower airway (LPAA). Both TB and MB reduced the severity of developing skeletal class II due to mandibular retrognathism by forward posturing of the mandible. Thus, patients with airway problems would benefit more from TB than MB.

## Data Availability

The datasets used and/or analyzed during the current study are available from the corresponding author on reasonable request.

## Abbreviations

TB: Twin block
MB: Myobrace
NPAA: Nasopharyngeal airway area
OPAA: Oropharyngeal airway area
LPAA: Laryngopharyngeal airway area
H: Harmonium
PNS: Posterior nasal spine. FHP:Frankfurt Horizontal plane
C5AI: Antero-inferior point of the 5th cervical vertebra
FMA: Frankfurt-mandibular plane angle
ICC: Intraclass Correlation Coefficient
SD: Standard deviation
CBCT: Cone-beam computed tomography
